# Cognitive mechanisms associated with auditory sensory gating

**DOI:** 10.1016/j.bandc.2015.12.005

**Published:** 2016-02

**Authors:** L.A. Jones, P.J. Hills, K.M. Dick, S.P. Jones, P. Bright

**Affiliations:** a Department of Psychology, Bournemouth University, Talbot Campus, Fern Barrow, Poole, Dorset BH12 5BB, UK; b Department of Neuroscience, Physiology, and Pharmacology, University College London, Gower Street, London WC1E 6BT, UK; c Department of Psychology, Anglia Ruskin University, East Road, Cambridge CB1 1PT, UK

**Keywords:** Sensory gating, Inhibition, Electroencephalogram, Event-related potential (ERP) P50

## Abstract

Sensory gating is a neurophysiological measure of inhibition that is characterised by a reduction in the P50 event-related potential to a repeated identical stimulus. The objective of this work was to determine the cognitive mechanisms that relate to the neurological phenomenon of auditory sensory gating. Sixty participants underwent a battery of 10 cognitive tasks, including qualitatively different measures of attentional inhibition, working memory, and fluid intelligence. Participants additionally completed a paired-stimulus paradigm as a measure of auditory sensory gating. A correlational analysis revealed that several tasks correlated significantly with sensory gating. However once fluid intelligence and working memory were accounted for, only a measure of latent inhibition and accuracy scores on the continuous performance task showed significant sensitivity to sensory gating. We conclude that sensory gating reflects the identification of goal-irrelevant information at the encoding (input) stage and the subsequent ability to selectively attend to goal-relevant information based on that previous identification.

## Highlights

• Sensory gating ratio negatively correlates with fluid intelligence.
• Sensory gating correlates with continuous performance and latent inhibition tasks.
• Sensory gating reflects identification and inhibition of irrelevant stimuli.
• Possible evidence for bottom-up and top-down influences on sensory gating.

## 1 Introduction

Sensory gating is a phenomenon in which the brain shows reduced evoked response to repeated stimuli (e.g., Boutros and Belger, 1999, Freedman et al., 1987, Freedman et al., 1996). It is typically explored using a conditioning-testing paradigm (or paired-stimulus paradigm) during an electroencephalogram (EEG) recording. The event-related potential (ERP) P50 is measured during the presentation of two identical stimuli with an inter-stimulus interval (ISI) of less than one second (Arnfred & Chen, 2004). The attenuation (’gating out’) of the P50 response to the second stimulus is the operational definition of sensory gating (Anokhin, Vedeniapin, Heath, Korzyukov, & Boutros, 2007) thought to be due to neural excitation of a set of sensory neurons to the presentation of the first stimulus generating P50, while simultaneously activating a second set of interneurons that inhibit any further excitatory response (Adler et al., 1985, Anokhin et al., 2007). This gives rise to a reduced response of the ERP P50 to the presentation of the second stimulus; the difference between these P50 responses indicates the strength of inhibition activated during the response to the first stimulus (Freedman et al., 2000).

A sensory gating deficit has been observed in several clinical populations. For example, patients with schizophrenia show reduced sensory gating compared to controls (Bramon et al., 2004, Patterson et al., 2008, Popov et al., 2011, Yee et al., 2010). Specifically, patient groups tend to have a gating ratio ranging from 56% to 158%, demonstrating an augmented response in some cases, while controls have a ratio of 9–73.4% (Patterson et al., 2008). This reduced sensory gating has been associated with impaired performance on tasks measuring sustained attention (Potter, Summerfelt, Gold, & Buchanan, 2006), latent inhibition (Lubow, Kaplan, Abramovich, Rudnick, & Laor, 2000), and inhibition of distractors (Erwin, Turetsky, Moberg, Gur, & Gur, 1998), and may be a core symptom of schizophrenia (Venables, 1964). The phenomenon is also observed, although to a lesser degree, in psychometric schizotypy (Fonseca-Pedrero, Lemos-Giraldez, Paino, & Muniz, 2011) as measured by the Schizotypal Personality Questionnaire (SPQ, Raine, 1991). However, more recent research has found no association between the sensory gating deficit in patients and their performance on neurocognitive tasks (Sánchez-Morla et al., 2013).

Although there is some evidence that sensory gating is a core deficit in schizophrenia and related to the endophenotype, cognitive correlates of sensory gating have not clearly been established. It is prudent to first establish a comprehensive understanding of sensory gating before it can be considered an endophenotype. The principal cognitive dimension that has been correlated with sensory gating is attention (Yadon, Bugg, Kisley, & Davalos, 2009). Attention encompasses a wide array of processes, including both stimulus selection and inhibitory mechanisms (Knudsen, 2007), and although sensory gating is thought to reflect the inhibitory processing component of attention, heterogeneity amongst standard tests of inhibition suggests this is a broad concept (Friedman & Miyake, 2004). Indeed, evidence for strong correlations between standard tests of inhibitory control is limited (Kramer et al., 1994, Shuster and Toplak, 2009, Yadon et al., 2009). Thus it is more constructive to focus on the several specific tasks and processes associated with sensory gating, rather than one task representing the broader trait of attentional inhibition. This is important to clarify those aspects of inhibition most closely related to sensory gating so that we can better understand the endophenotype of schizophrenia. Additionally, the inclusion of additional cognitive tasks may help identify or rule out the involvement of non-inhibitory mechanisms. The ability to isolate specific task effects is often complicated by a failure of published studies to adequately describe or identify the possible underlying mechanisms employed during task preparation and/or execution (Friedman & Miyake, 2004).

Inhibition is a form of cognitive control that functions to limit the processing of information in our environment (Frith, 1979). Harnishfeger (1995) characterises it in terms of three dichotomous dimensions: resistance to interference which gates information from entering working memory or the inhibition of information within working memory; intentional or automatic; and behavioural or cognitive (see also Nigg, 2000). These forms of inhibition may occur at different stages of information processing (e.g., Friedman & Miyake, 2004). Resistance to interference encompasses the ability to prevent the processing of task-irrelevant information in order to manage the relevant information in working memory more efficiently. One stage at which this can occur is the encoding (input) stage of information processing during which initial analysis and the selection of relevant stimuli is implemented. Following this, cognitive inhibition refers to the mental process of suppressing, or potentially eliminating, no longer relevant information that has already been encoded at an earlier stage, thereby precluding competition with information that is still regarded as relevant, though this might be confounded with response inhibition (MacLeod, Dodd, Sheard, Wilson, & Bibi, 2003). Finally, behavioural inhibition occurs at the output stage when a response is to be made. Consequently, if there is response conflict or a prepotent response that is maladaptive, behavioural inhibition is required to prevent incorrect responses and resolve conflict. Note that any measure of interference control encompasses all three proposed stages of processing, therefore conflating competition amongst multiple stimuli at the perceptual stage, processes at the cognitive stage, and responses during the behavioural output stage (Harnishfeger, 1995).

Nigg (2000) also identified three distinct forms of inhibition: executive, motivational and automatic. The current study employs Nigg’s distinction between executive and automatic inhibition, but does not include assessment of motivational inhibition, which is associated with emotional processing and is thought to reflect distinct neurological systems (Nigg, 2000).

### 1.1 Executive inhibition: interference control

Interference control is defined as the ability to suppress unwanted information from affecting performance (Nigg, 2000). The Stroop task (Stroop, 1935) is the classic measure of interference control, involving focused attention and response competition (Kok, 1999). A typical Stroop paradigm uses compound stimuli of colour words in which the colour of the ink and the word itself can be congruent (e.g., a blue word in blue ink) or incongruent (e.g., a blue word in red ink) (Lu & Proctor, 1995). Participants must identify the colour of the ink and therefore necessarily inhibit the more automatic response tendency of reading the word during presentation of incongruent trials. Longer reaction times are typically observed in these trials relative to the congruent trials (MacLeod, 1991).

A key feature of the Stroop effect is that of spatial integration: the task-irrelevant and task-relevant components of the Stroop are spatially integrated. This may enhance any possible conflict (Kaplan & Lubow, 2011), a claim supported by the observation that when the relevant colour and irrelevant word are perceptually separated using a time delay, the usual Stroop interference effect is significantly smaller (e.g., Dyer & Severance, 1973). Furthermore, when the word and colour are spatially separated but still presented simultaneously, there is typically no longer an interference effect (e.g., Flowers & Stoup, 1977).

A task that also measures interference control is the flanker task (e.g., Kaplan & Lubow, 2011). The flanker task measures the effects of irrelevant spatially separate distractor stimuli on the identification of a target stimulus. In general, the target stimulus appears in the centre of the visual field with distractor stimuli flanking it. As a result of the spatial arrangement, this task requires no visual search (Kaplan & Lubow, 2011). Flankers tend to slow response times to identify the target centre stimulus compared to when it appears alone (Eriksen & Hoffman, 1972). If these flankers are congruent with the target, response competition (Yücel et al., 2002) is minimal and response times are faster than if these flankers are incongruent with the target (Eriksen & Eriksen, 1974). Incongruent distracters interfere with the desired response, and/or activate the opposing response in working memory (Ridderinkhof, van den Wildenberg, Segalowitz, & Carter, 2004). In such tasks where the irrelevant and relevant information is presented simultaneously, the ability to focus attention on the target is required in order to allocate processing resources to the designated or relevant information (Kok, 1999).

A similar task that affects response-selection and comes under executive inhibition is that of the Simon task. Like the Stroop task, the Simon task is assumed to measure the ability to inhibit a response to and actively ignore a task-irrelevant stimulus dimension (Simon & Rudell, 1967). When a response to a particular dimension of a stimulus is made, the ability to make this response is affected by the relative spatial location of that stimulus to the response (Craft and Simon, 1970, Hommel, 1993). Faster responses are made to trials in which the task-irrelevant location of the stimulus is congruent with the location of the response (right visual field responded to by the right hand). Slower response times reflect a failure to inhibit a response to the task irrelevant dimension of the stimulus.

Yadon et al. (2009) found that the Stroop effect negatively correlated with the P50 gating ratio. Considering that the P50 is widely assumed to reflect inhibition, it is counterintuitive that a positive correlation was not found. Similarly, small and non-significant correlations between performance on the flanker task and sensory gating are typically found even though both tasks are proposed to measure a similar inhibitory deficit (Yadon et al., 2009). These results complicate theories linking sensory gating with cognitive interference, although further evidence based on other tests (e.g., the Simon test) should help confirm whether or not a relationship exists.

### 1.2 Executive inhibition: cognitive control

The ability to hold an item in working memory and subsequently ignore it is a typical process associated with inhibition (Nigg, 2000). This process is measured by the latent inhibition paradigm, in which participants are pre-exposed to a class of stimuli while ignoring another class of stimuli. This serves to promote implicit learning of the ignored set of stimuli (Rascle et al., 2001). In a subsequent task, the pre-exposed non-target stimuli become the target stimuli (Cohen et al., 2004, Lubow and Gewirtz, 1995). However, due to these stimuli having been non-targets, performance on the subsequent task is poorer than in the pre-exposure task and compared to novel stimuli (Braunstein-Bercovitz and Lubow, 1998, Escobar et al., 2002, Kaplan and Lubow, 2011). Latent inhibition refers to this inability to re-learn previously non-target stimuli as target stimuli (Granger, Prados, & Young, 2012). A simple explanation for the effect is that the previously non-target items have been given low attentional weights and it takes time to overcome this (Cohen et al., 2004, Lubow and Gewirtz, 1995, Pearce and Hall, 1980).

In the latent inhibition task, there is a concern that the critical test stimulus may just be eliciting a novel pop-out as a result of novel stimuli attracting more attention than old stimuli (Johnston & Hawley, 1994). Consequently, latent inhibition may actually reflect a facilitation effect for the non-pre-exposed stimuli rather than a deficit in performance for the pre-exposed stimuli. In response, Lubow and Kaplan (1997) created a method in which latent inhibition and the novel pop-out effect could be measured independently. They found that reaction times were always faster for novel stimuli compared to old stimuli regardless of whether the old stimulus was familiar due to previously being the target or non-target. This implies that latent inhibition likely reflects a pre-exposure performance deficit due to a reduction in attentional allocation, in addition to a non-pre-exposure facilitation effect due to novel pop-out when the non-target stimulus has not previously been presented.

Another task that purportedly measures cognitive control is that of negative priming. Each trial of a typical negative priming task consists of a prime display followed by a probe display (Fox, 1995). During test conditions, the prime display will contain a distractor that the participant is explicitly told to ignore (Beech, McManus, Baylis, Tipper, & Agar, 1991). This contrasts with latent inhibition in which participants tend to learn through experience to ignore the distractor. The target during the probe display can be novel, the target from the prime display, or the distractor from the prime display (e.g. Park, Puschel, Sauter, Rentsch, & Hell, 2002). During a typical negative priming task, response latency during the probe display is longer when it is preceded by a prime display in which the distractor became the target (Moritz & Mass, 1997). During the prime display the representation of the distractor or the response to it is suppressed (inhibited), and thus when this distractor becomes the subsequent target, it is harder to reactivate that representation resulting in longer reaction times (Aron, 2007, Tipper, 2001). Conversely, if the prime display distractor is not suppressed (remains activated), it will facilitate responding during its presentation as the target in the probe display (positive priming; Dux & Marois, 2008). However, this effect can be long lasting (DeSchepper & Treisman, 1996), possibly indicating the involvement of episodic memory retrieval rather than inhibition (Neill, 1997). Thus, the conflict generated during incongruent prime and probe displays is due to the probe display failing to match up to the memory representation of the prime display (Logan, 2002, MacLeod, 2007, Tipper, 2001).

### 1.3 Executive inhibition: intentional motor inhibition

Deliberate inhibition of a primary motor response to changing contextual cues is best demonstrated by the go/no-go task (Nigg, 2000). In this task, participants are required to make a response to a target stimulus and inhibit their response to a less frequently presented ’stop’ stimulus (Kok, 1986). The more frequent ‘go’ stimuli cause the action of responding to become a prepotent response. This task involves sustained attention in addition to response control, as participants need to pay attention to both the target and the ’stop’ stimuli, which do not appear simultaneously.

There are two proposed forms of response control, reactive and proactive (Aron, 2011). The former is a common form of response control in many inhibitory tasks including the go/no-go task. This reactive mechanism is initiated by the stop signal. It is the intentional inhibition of a currently activated response or goal as a result of new information (Nigg, 2000). Proactive inhibition is a way of anticipating the need to terminate a response before that need arises. This happens when a possible conflict is detected and all responses are paused until information regarding the next required response is provided. This can be illustrated in studies that compare randomised go/no-go trials with blocks of each type of trial. During randomised trials, participants are slower at responding but accuracy is not affected. This demonstrates a preemptive slowing of response in anticipation of a possible change in response type (Bogacz, Wagenmakers, Forstmann, & Nieuwenhuis, 2010).

A positive correlation has been observed between stopping error on the go/no-go task and P50 sensory gating (Yadon et al., 2009), which may suggest that sensory gating is related to intentional motor inhibition at the response stage of the task. This interpretation would refute the claim that sensory gating is a pre-attentive process due to the requirement to attend to stimuli and select the appropriate response. However, this correlation may reflect the sustained attention element of the go/no-go task and therefore support the opposite interpretation that sensory gating is pre-attentive. Issues with interpreting these results highlight the need for multiple tasks in determining the associated mechanisms with sensory gating.

### 1.4 Executive inhibition: oculomotor

Many of the executive inhibition tasks described above involve language or motor responses. There are tasks that involve simple ocular reflexes such as the antisaccade task in which participants must inhibit a reflexive response to the presentation of a stimulus. A typical antisaccade task requires the participant to move their gaze in the opposite direction to a presented stimulus (Hutton & Ettinger, 2006). In order to do this successfully, participants must inhibit the prepotent oculomotor response of directing their gaze towards a newly presented stimulus. The average error rate with this task, due to participants making a reflexive prosaccade, is around 20% (Ettinger et al., 2003). With regards to sensory gating, a correlation has been found between antisaccade performance and sensory gating (Cadenhead, Light, Geyer, McDowell, & Braff, 2002). Others suggest that the two forms of inhibition are independent of each other, with sensory gating being a largely automatic, pre-attentional cognitive mechanism and oculomotor inhibition, as measured by the antisaccade task, being effortful and dependent upon attention (Braff & Light, 2004).

### 1.5 Attentional orienting and sustained attention

Three separate anatomically and functionally defined attentional networks have been identified: orienting, alerting, and executive control (Fan et al., 2003, Posner and Petersen, 1990). Fan, McCandliss, Fossella, Flombaum, and Posner (2005) devised the Attentional Network Task (ANT) in order to assess these types of attention (Posner & Rothbart, 2007). The task involves a cued reaction time task and a flanker task, and the efficacy of each network is assessed by the reaction time differences between conditions. Each trial may have a cue or no cue, which provides either temporal or spatial information about the target. The target then appears above or below a fixation cross with congruent or incongruent flankers either side of it. The difference between congruent and incongruent trials is considered a marker of the efficiency of the executive functioning network (Flanker task), while the difference between a temporal cue and no cue is claimed to reflect alerting ability. Finally, the difference between the trials with and without an accurate spatial cue is intended to provide a measure for orienting proficiency. The orienting network controls the ability to focus attention towards the source of specific sensory signals by way of identification and selection of sensory stimuli (Posner & Rothbart, 2007).

Wan, Friedman, Boutros, and Crawford (2008) found a positive correlation between sensory gating and performance on the ANT. Those with superior gating capacity demonstrated greater accuracy and quicker reaction time predominantly in the alerting portion of the task, suggesting that sensory gating is related to attentional vigilance and precision. The alerting network has also been associated with the continuous performance and vigilance tasks. These tasks measure the capacity to remain alert over a long period of time (Fan, McCandliss, Sommer, Raz, & Posner, 2002) with the continuous performance task (CPT) directly measuring sustained attention (Nestor, Faux, McCarley, Shenton, & Sands, 1990). There are two main forms of this task, the CPT-single, which only requires the participant to respond when they see a target stimulus, and the CPT-AX or CPT-IP, which requires them to respond when they see the target stimulus but only when it is preceded by a cue stimulus (Lee & Park, 2006). Both variants of the task often take the form of a continuous stream of letters and participants respond when a pre-specified letter appears.

The ability to encode the relevant stimulus while ignoring the non-target stimulus, and maintaining the task instructions in working memory throughout the duration of the stream of stimuli is crucial to success on the CPT (Cohen et al., 1999, Oades, 2000). Additionally, in the CPT-AX forms of the task, the cue stimulus must be maintained in working memory. Participants must control what information is selectively attended to and similarly what information is excluded from working memory (Rush, Barch, & Braver, 2006). When patients with schizophrenia are selected on the basis of relatively high or low P50 sensory gating ratios, those with the higher ratios (i.e., with reduced sensory gating) typically perform worse at the CPT than those with lower ratios (Erwin et al., 1998), possibly due to increased susceptibility to distraction.

### 1.6 Top-down modulation of sensory gating

The notion of attentional influences upon sensory gating, primarily the early P50 response, is a contentious issue within the literature. Many researchers claim that the P50 response to auditory stimuli is a pre-attentive, automatic process, and thus unaffected by attentional manipulations (Boutros et al., 2004, Braff and Light, 2004, Freedman et al., 1991, Jerger et al., 1992). Any such effects are not observed until later processing, which is reflected in the component N100 (Braff and Light, 2004, White and Yee, 1997). However others have suggested that even components as early as P50, either the gating ratio or amplitudes, can indeed be affected by altering the capacity for sustained attention, or by directing attention towards the stimuli (Gjini et al., 2011, Rosburg et al., 2009, Yee et al., 2010). The effects of attention on P50 may reflect top-down processing of sensory stimuli working simultaneously with the bottom-up processes (Posner, 2004). Support for top-down influences on sensory gating comes from research with patients and animals with lesions to the pre-frontal cortex. This research has demonstrated that pre-frontal cortex damage impairs the ability to inhibit sensory information, specifically the ability to attend to relevant over irrelevant stimuli (Knight et al., 1989, Rosenkranz and Grace, 2001). Furthermore, developmental changes within the prefrontal cortex that lead to changes in attentional control may result in sensory gating improvements with age (Knight et al., 1999, Marshall et al., 2004). Further support for top-down influences on sensory gating has emerged from ERP studies which have found significant correlations between measures of frontal lobe dysfunction and sensory gating (Boutros et al., 2009), as well as P50 generators within the frontal lobes (Grunwald et al., 2003, Korzyukov et al., 2007, Liu et al., 2011, Mears et al., 2006).

## 2 The present study

In order to explore the cognitive functionality of sensory gating and provide a more comprehensive review of the possible underlying components, this correlational study tests participants’ sensory gating alongside their performance on each of the tasks described above. We predicted that sensory gating would correlate with several measures of inhibition, although detailed predictions could not be made with confidence due to unresolved issues in the literature. In this study, we have also assessed working memory and fluid intelligence in order to control for and explore potential top-down influences on sensory gating. Additionally, tests such as the CPT place substantial demands on working memory, raising the possibility that exploration of individual differences in psychometric intelligence may enable clearer distinction between task specific and more general cognitive factors associated with performance.

## 3 Method

### 3.1 Participants

An opportunity sample of 60 people (22 male, aged 19–30, mean = 21 years) participated in this study^1^. All participants self-reported that they had normal or corrected vision and hearing and were excluded if they had a history of brain damage, epilepsy, and/or alcoholism or were taking medication affecting the central nervous system at the time of participation. For the EEG investigation participants were asked to avoid alcohol 24 h prior to testing and were encouraged to refrain from smoking immediately before the session.

### 3.2 Design

A correlational design was employed whereby sensory gating ratios (see EEG analysis) were correlated with scores on each of the tests of inhibition (Stroop, Simon, latent inhibition, negative priming, go/no-go, switch, antisaccade, attentional network, and continuous performance). IQ (measured using Cattell’s Culture Fair (CCF-IQ) Scale 2 form A, Institute for Personality and Ability Testing, 1973) and working memory capacity (measured using the automated-OSPAN; Unsworth, Heitz, Schrock, & Engle, 2005) differences were controlled for by conducting partial correlations. Participants completed the tasks in a pseudorandom order (the exception to full randomisation being that the switch task always immediately followed the go/no-go task).

### 3.3 General procedures

Participants completed a series of cognitive tasks and an EEG recording. These were all conducted in dedicated, air-conditioned laboratories. Participants were tested one at a time and completed the tasks over 2-4 days in order to avoid fatigue. For each computer task (with the exception of the paper based IQ test (CCF-IQ)), participants were sat approximately 60 cm in front of a high-resolution 17″ LCD colour monitor. Most tasks were conducted on a Dell PC running E-Prime Professional 2, except for the Simon Task and the ANT which were run on a 15″ Toshiba LCD laptop running Psychology Experiment Building Language (PEBL) software (Mueller, 2013). The EEG task employed a dedicated response pad. All other task responses made by participants were recorded via a standard computer keyboard. All letters (block capitals), fixation stimuli, and arrow stimuli were black, bold, and in font courier new size 18, and were presented on a white background, unless otherwise specified. Practice trials for all tests were administered according to published guidelines (where available). Where an appropriate number of practice trials was not specified, 10 practice trials were administered.

### 3.4 Sensory gating procedure

Participants sat in a dimly lit, sound attenuated Faraday cage. The electrode cap was fitted using a chinstrap and/or chest strap depending on the fit. Participants were instructed to minimise any body, face, and eye movements. They were provided with an example of the auditory stimuli to be used in the experiment and the volume was adjusted to ensure a comfortable volume for all participants. Stimulus volume was first set at 75 dB using an SPL meter for all participants but was reduced marginally if participants found this volume too uncomfortable. During recording, they were monitored for signs of drowsiness by visual and EEG monitoring. Each trial consisted of a conditioning stimulus and a test stimulus that were both beeps: bursts of 4100 Hz of approximately 10 ms duration and an intensity of 70–75 dB, delivered using headphones. Each stimulus was presented for approximately 10 ms with an inter-stimulus interval (ISI) of 500 ms. There was an inter-trial interval of 10 s in order to allow brain activity to return to baseline (Sánchez-Morla et al., 2013). The experiment consisted of 135 paired-beep trials^2^ with an additional 15 click trials (4 ms bursts of 4100 Hz with an intensity of 70–75 dB) and was approximately 30 min long with two breaks. Participants were instructed to focus on a central black fixation cross on a white background throughout the experiment and to press the response pad whenever they heard a click. The primary reason for this task was to engage the participants. Subsequent analysis revealed all participants had an accuracy of at least 90%.

#### 3.4.1 EEG recording and data analysis

ERPs were recorded via two 32-channel DC amplifiers, using Brainvision Recorder and ActiCap software. Thirty-eight electrodes were mounted on a cap while four additional ocular electrodes were placed on the outer canthi and above and below one eye, which monitored horizontal (+HEOG, −HEOG) and vertical (+VEOG, −VEOG) eye movements respectively. Electrode impedances were kept under 10 kΩ when possible but were accepted when below 20 kΩ^3^. The sampling acquisition rate was 2000 Hz. FCz was the reference electrode during acquisition; the data were re-referenced to the average mastoids (TP9 and TP10) offline.

Using Brainvision Analyser, raw data were filtered using a 50 Hz notch filter and a 10–50 Hz band pass filter (24-dB/octave roll-off) (Boutros et al., 2004). Ocular corrections were made using a Gratton and Coles algorithm before semi-automatic data inspection was used to highlight and remove amplitudes exceeding −50/50 μV or gradients exceeding 50 μV/ms. For each participant, segmentations were made based on marker position for stimulus 1 (S1) and stimulus 2 (S2), which included 100 ms prior to stimulus onset and up to 200 ms post stimulus onset. Baseline corrections were carried out 100 ms before stimulus onset; this was completed before the averaging process was completed. Based on previous studies, the analysis focused on the P50 ERP within a 40 ms period between 40 ms and 80 ms post stimulus onset for electrode Cz. P50 amplitude was obtained by calculating the difference between the P50 peak and the preceding negative trough (N40). N40 was identified as the most negative peak between 20 ms and 50 ms post stimulus onset. Based on Gjini, Arfken, and Boutros (2010), if no obvious S1 P50 was observed, the participant was removed; however if no S2 P50 was observed then this was deemed to be full suppression and a value of 0.01 μV was given. For each participant, the P50 amplitude for Cz was obtained for S1 and S2. This procedure resulted in the removal of 9 participants (final sample size = 51).

**Fig. 1:**
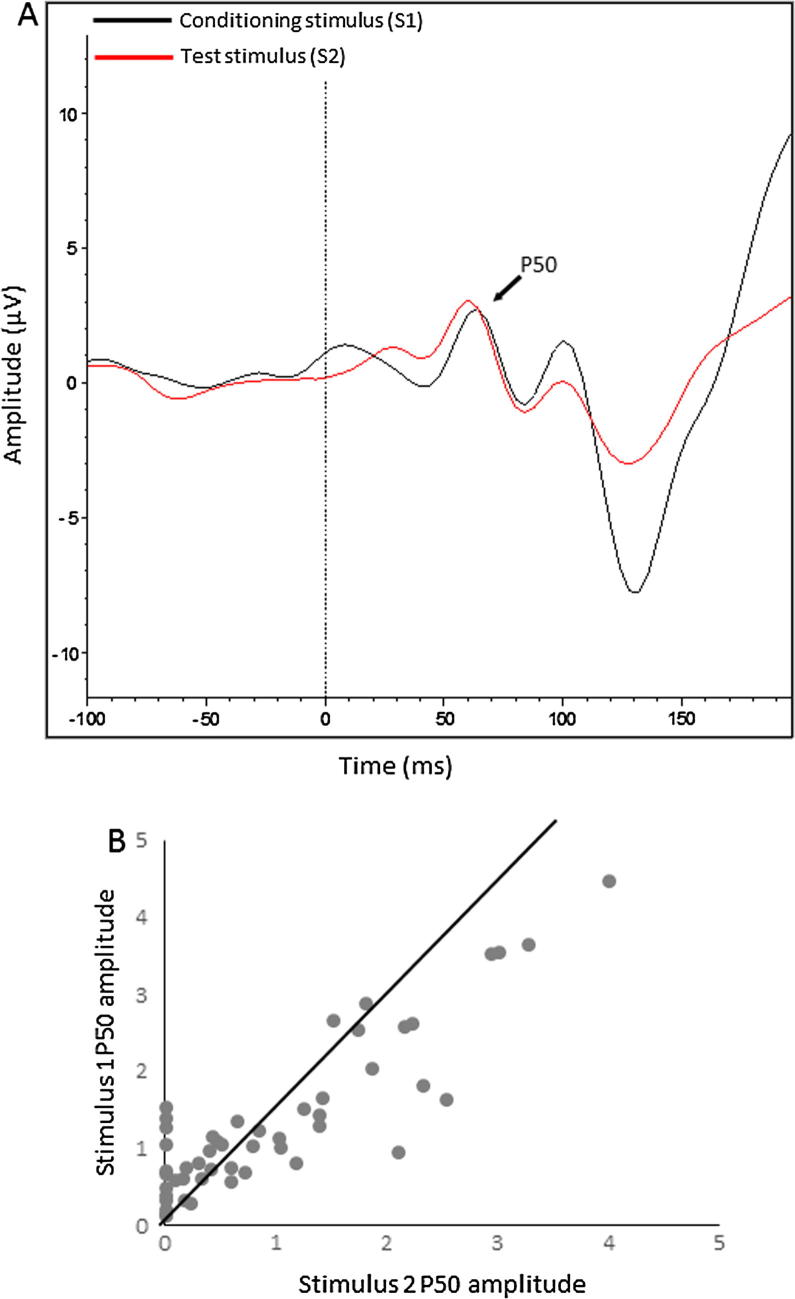
Panel A. Grand average EEG waveform in response to the presentation of the first and second stimulus at electrode Cz. Panel B. Scatterplot displaying the relationship between the P50 amplitudes for stimulus 1 and 2. The 45° line depicts the point at which there is no difference between the amplitudes. Points below the line illustrate individuals who demonstrated an attenuated P50 response to stimulus 2.

A sensory gating measure was established by calculating the P50 ratio (i.e., dividing the average P50 amplitude for S2 by the average P50 amplitude for S1 (S2/S1 ratio) and multiplying by 100). A smaller ratio (<100) is indicative of intact sensory gating. Any ratio above 200 was truncated to 200 to avoid outliers having a disproportionate effect on the analysis (Gjini et al., 2011). A paired-samples *t*-test confirmed that the amplitudes for the first stimulus (mean = 1.33 μV, SE = 0.14) was significantly larger than the amplitude for the second stimulus (mean = 0.97 μV, SE = 0.14) (*t*(49) = 5.1, *p* < .001), which illustrates a group level sensory gating effect (see Fig. 1A for the grand average waveform for stimulus 1 and 2, and Fig. 1B for the individual distribution of the P50 response to stimulus 1 and 2).

### 3.5 Behavioural inhibition tasks^4^

#### 3.5.1 Stroop task

Participants were informed that they were going to be presented with a series of words and asked to identify the ink colour of the word as quickly and accurately as possible. Response keys were coloured with all featuring colours (blue, green, purple, red, and yellow), and all words were written in block capitals, Courier New font size 18. For each trial the presentation of the colour word was terminated when a response was made. There were 30 trials that lasted approximately 1 min. During each trial the colour word could be the same colour as the ink (congruent) or a different colour to the ink (incongruent). The Stroop effect was operationalised by the difference in response times between congruent and incongruent trials (incongruent–congruent).

#### 3.5.2 Simon task

During each trial a fixation cross first appeared in the centre of the screen for 800 ms. Following a blank screen for 250 ms, a red or blue circle appeared either to the left or to the right side of the cross (visual angle 17.06°) for a maximum of 1 s if there was no response, or until the participant made a response. Participants were instructed to press the left shift key when they saw a blue circle and the right shift key when they saw a red circle, with the location of the stimulus treated as irrelevant. Half the trials were congruent (stimulus location corresponds to the response key) and the other half incongruent (stimulus location is opposite to response key). One hundred and fifty trials were presented in a random order and the task lasted approximately 2 min. For each participant, the Simon effect was operationalised as the difference in average reaction time between congruent and incongruent trials (incongruent–congruent).

#### 3.5.3 Latent inhibition

Stimuli consisted of five randomly connected straight black 1 cm lines on a white background subtending 10° of visual angle. Four designs were created. These were presented in an array containing 20 identical stimuli (target-absent condition), or 19 identical and 1 unique (target-present condition). Participants were instructed to identify whether there was a unique element within each array. The position of each stimulus was randomly generated in an imaginary 8 × 12 matrix. Participants were presented with 100 pre-exposure trials (50 target-present and 50 target-absent) in a random order. Only two of the stimuli were used in the pre-exposure phase (this was counterbalanced across participants). Stimuli remained on screen until the participant made a response. The test phase began immediately following the pre-exposure phase and participants were informed that they would be completing the same task but the stimuli would change from trial to trial. The duration of this experimental session was approximately 8 min.

There were seven possible relationships between the distractors and targets from the pre-exposure phase to the test phase, as described in Lubow and Kaplan (1997). However, only three are relevant to this study: (1) target and distractor stimuli in the pre-exposure phase swap roles in the test phase (PE); (2) target in the pre-exposure phase became the distractor in the test phase, and the test phase target was novel, assessing the novel pop-out effect (NPE); (3) both the target and the distractor in the test phase were novel (NOV). The average reaction time was recorded for each participant in each of the three main conditions. The effect of latent inhibition is measured by the reaction time difference in the PE condition minus the NPE condition. A novel pop-out effect is indexed by the NPE condition minus the NOV condition. Stimuli were counterbalanced across conditions.

#### 3.5.4 Negative priming

Participants were required to respond to a target symbol, ‘O’ and ignore a distractor symbol (‘X’) that appeared in one of four corners of a virtual square, subtended 0.6 × 0.6 degrees of visual angle. The location of the target symbol was identified by pressing ‘D’ indicating top left, ‘C’ indicating bottom left, ‘K’ for top right and ‘M’ for bottom right on a keyboard (corresponding to the location on the screen). Each trial consisted of a prime and probe display. In each trial, the prime and probe displays remained on screen until a response was made. Each screen was followed by a grey mask lasting 1350 ms. There was an inter-trial interval of 8–10 s. A fixation cross appeared in the centre of the screen 800 ms before each display to prepare participants. There were a total of 75 trials, which lasted approximately 18 min. There were three conditions: 1. both the target and distractor were in different places from the prime to the probe display (control); 2. the target in the probe display was in the same location as the distractor in the previous prime display (ignored-repetition); 3. no distractor was used (neutral). A negative priming effect is indicated when participants take longer to respond during the probe display of the ignored-repetition trials compared to the control trials (ignored repetition–control).

#### 3.5.5 Go/no-go and switch procedures

Participants were required to respond to a series of arrows based on the subsequent appearance of a target letter. All stimuli were white and presented centrally (visual angle 0.8°) on a black background. Participants were asked to produce speeded responses with the left or right index finger, according to the direction of the arrow presented prior to the target letter, ‘<’ corresponding to the left hand and ‘>’ corresponding to the right hand. They were instructed only to make a response when the letter ‘Z’ appeared (go trials, 80%), and withhold the response when the letter ‘T’ appeared (no-go trials, 20%). Visual feedback was presented immediately after a participants’ response. For each trial, the arrow was presented for 750 ms, followed by the letter (either target or non-target) for 1.5 s, with an inter-stimulus interval of 500 ms. There was an inter-trial interval of 1000 ms between each trial. The experimental session had 60 trials and lasted for approximately 4 min. The ability to withhold a response was represented as the number of errors made during no-go trials.

This procedure was immediately followed by the switch task. This was identical to the go/no-go task except that when ‘T’ was presented participants had to respond with the opposite hand and when ‘Z’ appeared they had to respond with the same hand as indicated by the direction of the arrow. The experiment session lasted for approximately 6 min and consisted of 100 trials. A response deficit for switch tasks is operationalised by the difference in response times between switch trials and repetition trials (switch–repetition).

#### 3.5.6 Antisaccade task

Participants were required to fixate on a central fixation cross for 200 ms before the trial commenced. The central fixation cross then illuminated green to indicate a pro-saccade trial (to look at the target) or red to indicate an anti-saccade trial (to look at the mirror location to the target). At the same time, a laterally displaced target (either 8° to the left or right of the centre) appeared. This remained on screen until the participant made a correct saccade, as recorded by the Tobii 1750 eye-tracker. The central fixation cross disappeared after 200 ms of fixation at the target. There was a random inter-trial-interval of between 2000 and 2500 ms. The targets were presented in white on a black background. The time to make the correct saccade was measured. Participants completed a total of 180 trials (divided equally amongst pro- and anti-saccade trials of which half involved a target presented to the left and half involved a target presented to the right). The task measures the ability to inhibit a pre-potent response, and is indexed by the difference in reaction time to make anti-saccades compared to pro-saccades.

#### 3.5.7 Attentional Network Task (ANT)

During this task all stimuli were presented in white on a black background. Each trial consisted of a fixation cross in the centre of the screen for 400–1600 ms, followed by a second fixation period that could contain a cue stimulus in the form of an asterisk for 100 ms. In no-cue conditions, the fixation cross appeared alone for 100 ms. After the cue, there was a further 400 ms fixation period before the presentation of target stimuli. During the target stimulus display, there were a total of seven stimuli, three flankers either side of one target stimulus. The six flanker stimuli appeared for 50 ms. While the flankers remained on the screen, the target appeared in the centre for 100 ms, thus the flankers were on screen for a total of 150 ms. This target and flanker array would appear either above or below the fixation cross but with the target stimulus centrally aligned with the cross. The inter-trial interval was dependent upon the participant’s response time to the target stimulus and the variable time of the first fixation period. Participants were instructed to report the direction of the central arrow (visual angle 0.8°) amongst three flanker arrows on each side (visual angle: outside edge 4.9°, inside edge 2.2°) pointing either left (<) or right (>). Participants’ made responses by pressing the keyboard, ‘z’ for left and ‘m’ for right’. There were a total of 290 trials lasting approximately 30 min. Trials were presented in a random order.

The six flankers were identical and could either be the same as the target stimulus (congruent), the opposite stimulus (incongruent), or unrelated. Additionally, during the second fixation period when a cue could be presented, there were a further four conditions. Either no cue would be presented (no cue), a cue indicating the appearance of the target stimulus in the centre where the fixation cross was (centre cue), a cue appearing both above and below the fixation cross also providing temporal information (double cue), and a cue which would appear either above or below the fixation cross providing temporal information as well as spatial information by indicating where the target stimulus will appear.

Only correct responses were used to calculate the various attentional measures. The executive functioning component of this task (the flanker task) is indexed by a slower responding during incongruent trials compared to congruent (incongruent–congruent). The alerting component is operationalised by the difference in response time between the centre cue condition and the no cue conditions (no cue-central cue). Finally, the difference in response time between the trials with a spatial cue compared to no cue, provide a measure for orienting proficiency (no cue–spatial cue).^5^

#### 3.5.8 Continuous performance task

Each trial began by presenting a central fixation cross, participants’ initiated the start of the task by pressing the ’space’ key. Following this, letter strings were presented centrally for 250 ms each, with an ISI of 750 ms. Participants were required to press the ‘space’ key whenever they saw the target letter ‘X’. There were a total of 273 trials in which 30% were a target ‘X’. The experimental session lasted approximately 5 min. Average accuracy and response times were recorded for target trials.

### 3.6 Psychometric tasks

#### 3.6.1 Cattell’s culture fair measure of fluid intelligence

Participants completed the Cattell Culture Fair Intelligence Test Scale 2, form A (Institute for Personality and Ability Testing, 1973), which was used to assess individual differences in fluid intelligence. This IQ test has been widely used and has good construct and concrete validity scores, (.81 and .70 respectively); test-retest, internal and external reliability scores of .73, .76, and .67 respectively.

#### 3.6.2 OSPAN

Working memory proficiency was measured using the automated-OSPAN task (Unsworth et al., 2005). Participants were required to memorise letters while solving mathematical problems. This task consisted of mathematical stimuli such as “(3 × 2) + 4 = 11?” When an equation was presented, participants were instructed to click the mouse when they had solved the equation, which then brought up a single digit in the centre of the next screen. They then had to indicate whether the digit was correct by clicking a “true” or “false” box with a computer mouse. After each equation, a letter appeared and participants were informed that they would be asked to recall these letters at a later point in the same order in which they were presented. The number of compound stimuli (one mathematical followed by one letter) presented, before recall of the letters was required, varied from 2 to 7. When participants were required to recall the letters, 23 letters (correct and incorrect) were presented as a 4 × 3 matrix and participants had to click a box next to the appropriate letters in the correct order using the computer mouse. It was emphasised during the task that the mathematical problems must be answered correctly; feedback was displayed in red at the top right of the computer screen indicating the percentage of correct answers, which was kept above 85%. This task lasted approximately 10 min. The absolute OSPAN score is the number of correct letters remembered in the correct order but only from sets in which all letters were recalled correctly.

## 4 Results

**Fig. 2:**
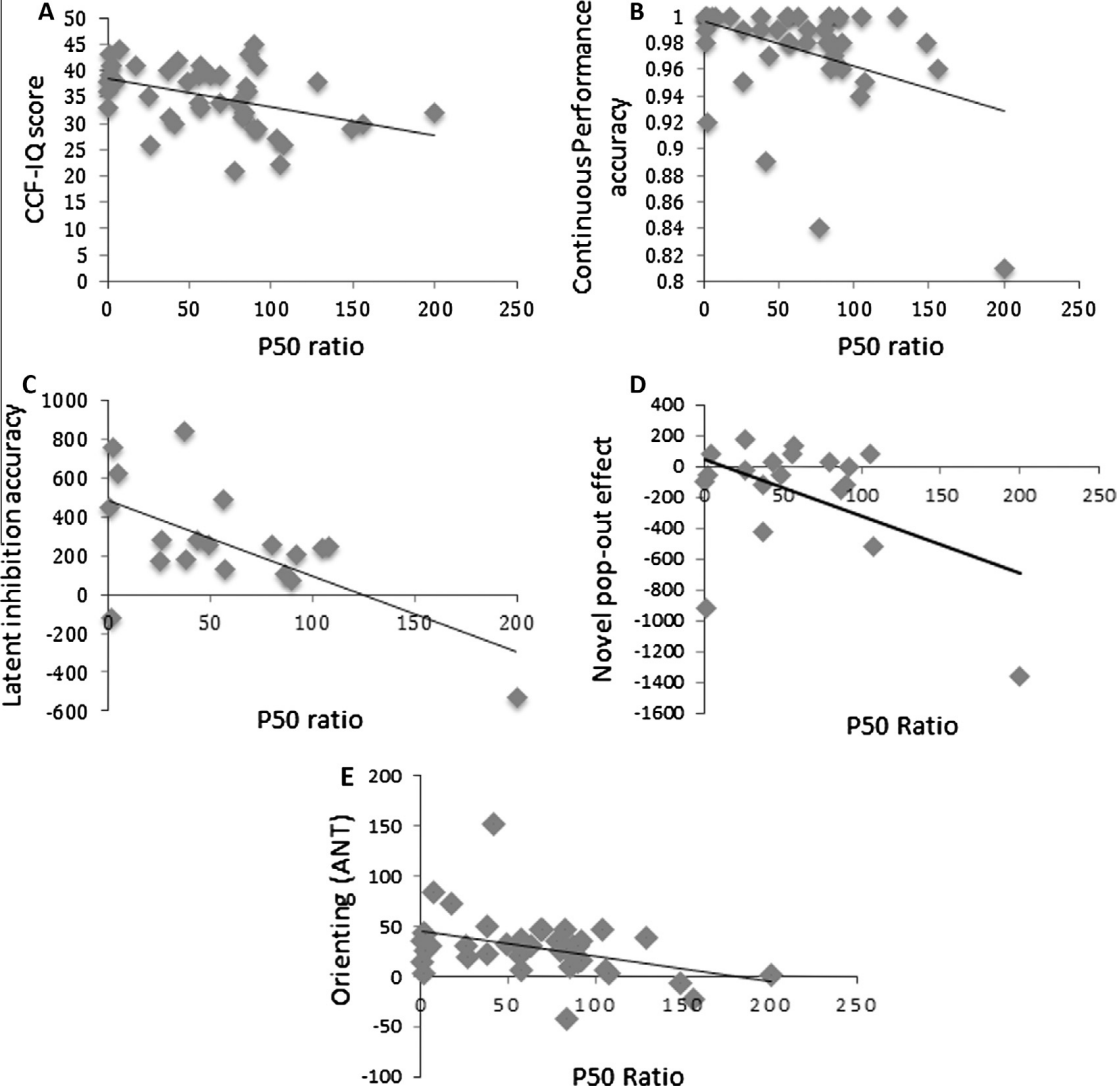
Scatterplots for significant correlations between cognitive performance measures plotted against the P50 ratio. Panel A. plots performance on the Cattell’s Culture Fair measure of intelligence (CCF-IQ; max raw score is 46). Panel B. shows accuracy on the Continuous Performance Task, panel C. displays Latent Inhibition accuracy, panel D. displays the novel pop-out effect, and panel E. displays the orienting component of the attentional network task. Each panel shows a trend line reflecting the negative relationship in each instance.

Using a bivariate correlation a significant negative correlation was observed between the sensory gating ratio and CCF-IQ performance, suggesting that successful sensory inhibition is related to fluid intelligence. Additional significant negative correlations^6^ were found between the sensory gating ratio and the latent inhibition effect (*r* = −.63), the novel pop-out effect (*r* = −.47), the orienting component of the attentional network task (*r* = −.38), and accuracy scores on the CPT (*r* = −.38) (see Fig. 2 depicting the correlations between sensory gating and CCF-IQ performance, CPT accuracy and latent inhibition). All other correlations were non-significant (*p* > .05). A summary of correlations between the attentional inhibition tasks and sensory gating can be seen in Table 2. While Fig. 2 may indicate the presence of outliers driving this correlation, we ensured that there were no outliers by exploring the standardised residuals and extreme values: no data point was considered an outlier (all standardised residuals were between −1.96 and +1.96). To measure the difference in magnitude of these correlations, a series of Williams’s *t*-tests were used following a Fisher’s *r* to *Z* transformation. These results show that none of the correlations significantly differed from one another *(p* > .05), indicating that the magnitude of the correlations between each of the five tasks and sensory gating were similar.

After statistically controlling for intelligence (as measured by CCF-IQ), correlations between the sensory gating ratio and both the novel pop-out effect and the orienting component of the attentional network task were no longer significant (*p* > .05). However, there remained a significant negative correlation between the sensory gating ratio and the latent inhibition effect (*r* = −.62), and the accuracy scores for the CPT (*r* = −.58). Again a William’s *t*-test was conducted to explore the magnitudes of these two correlations. Neither the latent inhibition effect nor the accuracy score during the CPT correlated to a different degree with the sensory gating ratio (*Z* = .15, *p* = .880).

Working memory (as measured by the OSPAN) was also controlled for, resulting in comparable findings to that of CCF-IQ. The correlations between sensory gating and both the novel pop-out effect and attentional orienting were no longer significant, while the correlations between sensory gating and both latent inhibition (*r* = −.63) and continuous performance (*r* = −.57) remained significant. Again there was no significant difference between the magnitude of the correlation coefficients for latent inhibition and orienting (*Z* = .22, *p* = .413). These results were again similar when both fluid intelligence and working memory were controlled for together. Changes in the correlation coefficient when controlling for either/both fluid intelligence or working memory, can be seen in Table 3.

## 5 Discussion

In the present study we explored the cognitive functionality of sensory gating, using auditory sensory gating and a battery of tests that tap different aspects of cognitive function. We predicted that sensory gating would correlate with several measures of inhibition. We demonstrate significant correlations between sensory gating and performance on the continuous performance and latent inhibition tasks. Correlations with other tasks, which also incorporated attentional inhibition, were weak and non-significant.

Higher sensory gating ratios were associated with reduced latent inhibition. Specifically, greater inhibition resulted in a larger detriment to performance when target stimuli in the test phase were non-targets during the pre-exposure phase. Latent inhibition occurs when repeated presentations of a stimulus without consequence results in that stimulus being regarded as task-irrelevant. Subsequently, identifying that stimulus as the target in the following test phase is more demanding (Braunstein-Bercovitz and Lubow, 1998, Escobar et al., 2002). A primary feature of latent inhibition is that of selective attention whereby participants preferentially process stimuli that are deemed relevant to the exclusion of all other stimuli. Thus, limited attentional resources are focused upon aspects of the environment that are salient to the current task demands and goals (Granger et al., 2012, Lubow, 1989). Essentially, the learned irrelevance must be overcome.

Evidence that the latent inhibition effect reflects processing of the non-target stimulus during the pre-exposure phase is supported when only the same stimulus at pre-exposure and test can elicit the latent inhibition effect, suggesting that some encoding of that particular stimulus (at pre-exposure) must occur (Lavie & Tsal, 1994). The encoding most likely reflects evaluation of stimulus relevance in order to prevent further attentional allocation to the non-target stimulus during the test phase.

Those with reduced latent inhibition (i.e., non-clinical high-psychotic participants) typically perform better on subsequent recognition and recall of the pre-exposed stimulus, despite a change in context (Beech, Baylis, Smithson, & Claridge, 1989). A change in context normally eliminates the effects of latent inhibition in typical subjects. . Taken together, this research suggests that latent inhibition is caused by both the encoding of the pre-exposed stimulus and the subsequent ability to inhibit that stimulus from further encoding through selective attention once it has been deemed task-irrelevant.

In addition to the relationship with latent inhibition, the present finding showed a sensitivity of sensory gating to accuracy on the CPT, with higher sensory gating ratio (indicative of reduced inhibition) associated with worse performance. This finding may reflect a failure of participants with higher gating ratios to attend to the relevant stimuli for sustained periods due to a difficulty in suppressing goal irrelevant distractor information. However, the single version of the task, as used in this study, is considered a measure of stimulus vigilance during sustained attention and encompasses limited, if any, suppression demand (e.g., Halperin et al., 1991, Nuechterlein and Dawson, 1984, van den Bosch et al., 1996).

The sensory gating and CPT procedures are structurally similar. Stimulus vigilance is measured in the single version of the CPT by presenting participants with an occasional target stimulus (to which they must respond) amongst a sequential stream of letters. Similarly, during the sensory gating task, participants were presented with a stream of identical auditory stimuli and were required to respond whenever an occasional different target stimulus was heard. The only obvious differences between the two tasks were that of different inter-trial intervals, input modality (which may indicate that sensory gating is not modality specific), and the use of identical rather than several different ‘no response’ stimuli in the sensory gating paradigm. The extent to which sensory gating is modality specific remains unresolved in the literature, but our findings are most consistent with a domain general view, in which gating operates across multiple input modalities. Nevertheless, further research will be required to resolve this debate. Putting aside the issue of input modality, both paradigms are closely similar in terms of task demands, and this conceptual similarity is likely to underpin the significant correlation between them. Additionally, even after the partial correlation in which CCF-IQ performance or the OSPAN score was the controlling factor, this correlation remained, which would be expected if they both entail similar task demands and additionally share some common underlying component independent of psychometric intelligence. Ceiling effects on the CPT limit the extent to which we can draw firm conclusions about the strength of the relationship with sensory gating, and the cognitive mechanism(s) underpinning both measures.

Negative priming did not correlate significantly with sensory gating, an unexpected finding given claims that it reflects the same underlying cognitive control mechanisms as latent inhibition (e.g., Nigg, 2000). This lack of correlation potentially provides support for the theory that negative priming does not reflect a component of inhibition but rather episodic retrieval processes (Neill, 1997).

Another difference between the negative priming effect and latent inhibition is the involvement of instruction. In negative priming, participants are informed about what is task-relevant and task-irrelevant prior to testing, whereas what is deemed task-irrelevant in the latent inhibition effect is ’learnt’ through repetition. This assertion suggests that latent inhibition reflects an encoding stage at which the assessment of relevancy and saliency is conducted. This is similar to sensory gating paradigms in which participants are not informed that the second of the two stimuli is irrelevant.

We propose, therefore, that sensory gating reflects the identification of context specific irrelevance at the encoding (input) stage that is governed in part by goal-directed processes, and/or the subsequent ability to selectively attend to relevant stimuli based on the previous identification. Further research will be required to confirm whether sensory gating reflects both or just one of these mechanisms. With respect to proposed ‘subcategories’ of inhibition (Harnishfeger, 1995, Nigg, 2000), sensory gating may be related to the categorisation of task-irrelevance at the input stage as well as selective attention at the cognitive processing stage (cognitive inhibition). Consequently, sensory gating requires an element of top-down as well as bottom-up processing in order for task demands to influence selective attention. Although sensory gating is an early process, occurring at 50 ms post-stimulus onset, it is possible that top-down influences are in operation. Research indicates that top-down networks may be activated even before the presentation of stimuli (Corbetta et al., 2000, Kastner et al., 1999), during the expectancy period, and may continue to be active during the presentation of stimuli (Kastner et al., 1999). It is this top-down processing that enables selective attention to relevant stimuli based on task demands (Pessoa, Kastner, & Ungerleider, 2003). The proposed neurological generators of sensory gating provide further support for these top-down influences. The fronto-temporal interaction model of sensory gating suggests that gating of basic stimulus properties may occur in the auditory cortex while additional or more goal-directed gating is more contingent upon prefrontal cortex (Jensen et al., 2008, Mears et al., 2006, Oranje et al., 2006, Tregellas et al., 2007). Consistent with this model, deficits in sensory gating have been observed in patients and animals with prefrontal lesions (Knight et al., 1999, Rosenkranz and Grace, 2001).

There was also a significant positive correlation between sensory gating and performance on CCF-IQ, a measure of non-verbal fluid intelligence (Cattell & Cattell, 1960). Research has suggested that correlations with intelligence may reflect the selection strategies employed during encoding (Cusack, Lehmann, Veldsman, & Mitchell, 2009). Those with higher intelligence encode the most relevant stimulus features. This is consistent with the proposed correlation between sensory gating and latent inhibition described above. Indeed intelligence is also related to top-down goal-directed processing during selective attention (Duncan, 1995), and both intelligence and goal-directed behaviour are associated with activation of a fronto-parietal network (e.g., Colom et al., 2010, Dumontheil et al., 2011, Duncan et al., 2008).

Several tasks failed to significantly correlate with sensory gating, including some measures of interference control, intentional motor inhibition, oculomotor inhibition, and attentional orienting. In the Stroop, Flanker, Simon, go/no-go, and switch tasks, there is a principal element of response competition and conflict resolution. Typically, there are either two opposing responses available, or there are stimuli that are incongruent with an automated/prepotent response, therefore requiring effortful overturning of that response tendency. In these tasks, then, the goal-relevant response occurs at the output stage of stimulus processing and may therefore be more related to behavioural inhibition than to the early processing reflected in sensory gating. This is not a feature of latent inhibition, as there are no competing responses or trials involving the congruency of target and response.

## 6 Conclusions

In summary, we observed significant correlations between sensory gating and i. latent inhibition and ii. accuracy on the CPT (but not other attentional inhibition tasks), after statistically controlling for differences in fluid intelligence and working memory. These findings suggest that sensory gating is associated with specific aspects of goal-directed attentional control. We propose that fundamental to sensory gating is the identification of goal irrelevant information such that attention to that information is reduced relative to goal relevant information. Both top-down and bottom-up processes that occur at the initial encoding stage of stimulus processing underpin this ability. Additionally, sensory gating enables resistance to interference as well as early cognitive inhibition at the encoding stage compared to other inhibition tasks that arguably involve more cognitive and behavioural inhibition at the output/response stage.

## Figures and Tables

**Table 1:** Test information and descriptive statistics for all cognitive control measures used in experiment.

Predictor variable	Test authors	Index and test of effect
*Executive inhibition: interference control*		
Stroop effect	Stroop (1935)	Mean incongruent RT (1009 ms, *SD* = 262) was greater than mean congruent RT (934 ms, *SD* = 211), *t*(48) = 5.32, *p* < .001
Simon congruency effect	Hommel (1993)	Mean incongruent RT (486 ms, *SD* = 94) was greater than mean congruent RT (458 ms, *SD* = 82), *t*(44) = 4.45, *p* < .001
Conflicting (ANT)	Fan et al. (2002)	Mean incongruent RT was greater than mean congruent RT (mean difference = 92.35 ms, *SD* = 65.38), *t*(44) = 8.85, *p* < .001

*Executive inhibition: intentional motor inhibition*		
No/No-Go Effect	Rubia et al. (2001)	Mean percentage accuracy for go trials (not-responding, 98%, *SD* = 4) was greater than mean percentage accuracy for no-go trials (responding, 85%, *SD* = 18), *t*(48) = 4.98, *p* < .001
Switch cost	Mean switch RT (513 ms, *SD* = 111) was greater than mean non-switch RT (454 ms, *SD* = 64), *t*(48) = 6.26, *p* < .001

*Oculomotor inhibition*		
Anti-Saccade Difference	Brenner, McDowell, Cadenhead, and Clementz (2001)	Mean RT to make anti-saccades (1738 ms, *SD* = 1440) was longer than mean RT to make pro-saccades (871 ms, *SD* = 492), *t*(47) = 4.50, *p* < .001

*Executive inhibition: cognitive control*		
Latent inhibition	Lubow and Kaplan (1997)	Mean RT on pre-exposed trials (1331 ms, *SD* = 456) was longer than mean RT on non-pre-exposed trials (1074 ms, *SD* = 492), *t*(20) = 3.76, *p* = .001
Negative priming effect	Park et al. (2002)	Mean RT on ignored repetition trials (574 ms, *SD* = 203) was greater than mean RT on neutral trials (490 ms, *SD* = 157), *t*(48) = 5.48, *p* < .001

*Sustained attention*		
Continuous performance accuracy	Lee and Park (2006)	Mean accuracy to respond to target stimulus (97%, *SD* = 6) was greater than chance, *t*(48) = 122.67, *p* < .001
Continuous performance RT	Mean time to respond to target stimulus was 523 ms (*SD* = 14)

Alerting (ANT)	Fan et al. (2002)	Mean double cue RT (584 ms, *SD* = 98) was greater than mean no cue RT (534, *SD* = 90), *t*(44) = 8.69, *p* < .001
*Attentional orienting*		
Orienting (ANT)	Fan et al. (2002)	Mean spatial cue RT (584, *SD* = 98) was faster than mean central cue RT (511, *SD* = 94), *t*(44) = 6.66, *p* < .001

*Covariates*		
CCF-IQ	Cattell and Cattell (1960)	Mean raw intelligence score was 35.12 (*SD* = 5.80)
Automated OSPAN	Unsworth et al. (2005)	Mean OSPAN absolute score was 39.06 (*SD* = 18.66)

*Note.* Only reaction times from trials with correct responses were used.

**Table 2:** Correlation coefficients from bivariate correlation between sensory gating and the measures of inhibition and psychometric tests.

Task correlations with sensory gating	Correlation coefficient (*r*)	*N*
*Executive inhibition: interference control*		
Stroop	.06	43
Simon	.07	39
Flanker/executive control (ANT)	.04	41

*Executive inhibition: cognitive control*		
Latent inhibition	−.63⁎⁎	19
Novel pop-out	−.47⁎	19
Negative priming	.18	43

*Executive inhibition: intentional motor inhibition*		
Go/no-go	−.16	43
Switch	.16	43

*Oculomotor inhibition*		
Antisaccade task	−.15	43

*Attentional orienting*		
Orienting (ANT)	−.38⁎	41

*Sustained attention*		
Alerting (ANT)	.03	41
Continuous performance	−.38⁎	43

*Covariates*		
Intelligence: CCF-IQ	−.42⁎⁎	43
Working memory: OSPAN	−.23	43

⁎ Sig. to .05.

⁎⁎ Sig. to .01.

**Table 3:** Correlation coefficients after partial correlation controlling for fluid intelligence (CCF-IQ) and working memory (OSPAN).

Task correlating with sensory gating	Bivariate correlation	Controlling for Cattell’s	Controlling for OSPAN	Controlling for Cattell’s and OSPAN
Correlation coefficient (*r*)	Correlation coefficient (*r*)	Correlation coefficient (*r*)	Correlation coefficient (*r*)
Latent inhibition	−0.63⁎⁎	−0.62⁎	−0.63⁎⁎	−0.63⁎
Novel pop-out	−0.47⁎	−0.48	−0.43	−0.45
Orienting (ANT)	−0.38⁎	−0.38	−0.42	−0.37
Continuous performance	−0.38⁎	−0.58⁎	−0.57⁎	−0.56⁎

⁎ Sig. to .05.

⁎⁎ Sig. to .01.

## Footnotes

1 Actual sample sizes are included per analysis in the Results section.

2 An average of 5% of trials were removed per participant due to noise in the data.

3 Impedances were accepted below 20 kΩ if the time to set up the EEG exceeded 40 min. The majority of the time impedances were between 0 and 3 kΩ and the EEG was visually inspected prior to recording to assess data quality.

4 Information regarding the authors of each methodology and the group-level test of effect can be seen in Table 1.

5 These measures are grouped by domain in Table 1, Table 2.

6 Correlations were significant after controlling for Type I error by applying the Bonferroni–Šidak correction for multiple comparisons.
